# Spatial localization of arachidonic acid in human carotid atherosclerotic plaques reveals a pro-inflammatory metabolic program in macrophages

**DOI:** 10.3389/fmolb.2026.1786539

**Published:** 2026-03-25

**Authors:** Jiaxin Wan, Rijin Lin, Zhouyang Jiao, Hui Cao, Chuang Zhang, Xiaowen Zhang, Mengyan Fan, Nan Zhang, Jiamei Zhang, Huixiang Liu, Yike Zhang, Chen Huang, Jianglin Yang, Jing Li, Jie Zhang, Sheng Guan

**Affiliations:** 1 Department of Neurointervention, the First Affiliated Hospital of Zhengzhou University, Henan Provincial Neurointerventional Engineering Research Center, Zhengzhou, Henan, China; 2 Department of Endovascular Surgery, The First Affiliated Hospital of Zhengzhou University, Zhengzhou, China; 3 Department of Emergency Medicine, The First Affiliated Hospital of Zhengzhou University, Zhengzhou, China; 4 Fujian Provincial Key Laboratory of Neurodegenerative Disease and Aging Research, Institute of Neuroscience, College of Medicine, Xiamen University, Xiamen, Fujian, China

**Keywords:** arachidonic acid, atherosclerosis, macrophage, single-cell sequencing, spatial metabolomics

## Abstract

**Background:**

Carotid atherosclerosis is a significant cause of ischemic stroke. It is a chronic inflammatory disease characterized by the progressive accumulation of inflammatory cells and mediators. Specific key metabolites are known to play pivotal roles in the progression of atherosclerosis. By applying spatial omics, we pinpointed the colocalization of arachidonic acid with inflammatory cells in plaques, providing direct spatial evidence for its pro-inflammatory role in atherosclerosis.

**Methods:**

We employed metabolomics, spatial metabolomics, and single-cell transcriptomics to compare human stable and unstable plaques, aiming to identify key molecules associated with atherosclerotic disease progression. The spatial distribution of key metabolites and lipid components was analyzed using matrix-assisted laser desorption/ionization mass spectrometry imaging, enabling a detailed description of their spatial characteristics within carotid atherosclerotic plaques.

**Results:**

Collected human carotid artery atherosclerotic plaque tissues via carotid endarterectomy (CEA) for metabolomic analysis, identifying 74 differential metabolites. Notably, the pro-inflammatory lipid arachidonic acid (AA) was involved in 22 of these pathways and was upregulated in unstable plaques. ROC curve analysis further indicated that AA had good predictive capability for the disease. Focused on investigating the metabolic processes of AA. Using spatial metabolomics technology, revealed the dynamic spatial distribution of the “linoleic acid–AA–leukotriene D4” metabolic axis within atherosclerotic plaques. Based on the pro-inflammatory properties of AA, further explored its spatial distribution within plaques and its association with macrophages. Through single-cell sequencing analysis of macrophage subsets, found that *ELOVL5* and *ALOX5* were highly expressed in macrophages.

**Conclusion:**

Our study provides direct spatial evidence for the existence of the “linoleic acid-AA-leukotriene D4” metabolic axis. It reveals the association of *ELOVL5* and *ALOX5* with macrophage phenotypes, demonstrating their potential in regulating atherosclerosis-related inflammation.

## 1 Introduction

Ischemic stroke represents a major global public health challenge, characterized by high incidence, mortality, and disability rates (Feigin et al., 2009). Approximately 20% of ischemic strokes are attributable to atherosclerosis (Petty et al., 1999), this subtype, termed atherothrombotic stroke, carries a higher risk of recurrence compared to other subtypes. The pathogenesis primarily involves stenosis and plaque rupture caused by atherosclerosis. Plaque rupture can lead to local vessel occlusion by thrombus or trigger distal emboli, ultimately resulting in stroke. The progression from stable to rupture-prone plaque is continuous (Bentzon et al., 2014). The initial stage of carotid atherosclerosis is characterized by the formation of fatty streaks. Macrophages within the plaque derive from monocytes recruited to the intima during inflammatory responses. These monocytes differentiate into macrophages, which then uptake oxidized low-density lipoprotein (oxLDL) to become foam cells, constituting early atherosclerotic lesions (fatty streaks) (Libby et al., 2019). Consequently, these early plaques consist of layers of lipid-laden macrophages. Ultimately, the atherosclerotic plaque evolves into a heterogeneous and complex structure, potentially including calcification, a necrotic core, thrombus, and plaque rupture, culminating in a stroke event. Atherosclerosis is a complex chronic inflammatory vascular disease. Its hallmarks include the deposition of metabolites (particularly lipids) at lesion sites and the synergistic action of various inflammatory cascades that drive disease progression. Lipids and related metabolites play a pivotal role: they enter the arterial wall through damaged endothelium and are retained within the intima, where they participate in diverse immune and inflammatory response processes. The persistent inflammatory environment within the plaque worsens as the plaque grows (Goikuria et al., 2018).

Arachidonic acid (AA), an omega-6 polyunsaturated fatty acid (PUFA), consists of a 20-carbon chain with 4 cis double bonds. Among omega-6 fatty acids, linoleic acid (the shortest-chain omega-6 fatty acid and a precursor to AA) is one of the most significant PUFAs. Free AA serves as a substrate for various enzymes, generating a multitude of AA-derived metabolites with diverse functions. Components of the AA pathway have been identified as crucial mediators involved in the progression of metabolic diseases and are significant factors influencing both the initiation and resolution of inflammation (Sonnweber et al., 2018). In atherosclerosis, circulating AA and its metabolites are key regulators of inflammation and lipid metabolism. Elucidating the mechanisms of action of AA and its metabolites will provide valuable guidance for the preclinical and clinical management of atherosclerosis (Bäck et al., 2019; Hu et al., 2024). Although existing research indicates that AA and its metabolites play important roles in atherosclerosis, technological limitations have hindered the acquisition of accurate spatial distribution information. Given the significant correlation between the spatial distribution of lipids and plaque stability, the relationship between their spatial localization and pathological regions of the plaque still requires validation. Furthermore, the association between AA and pro-inflammatory cells warrants further investigation.

Spatial omics is an emerging omics technology that has garnered significant attention in recent years (Bressan et al., 2023). Spatial metabolomics, a branch of this field, focuses on the spatial distribution characteristics of small molecules, particularly important metabolites such as lipids. Leveraging advanced imaging technologies and analytical methods, spatial metabolomics enables the visualization of the spatial localization and relative abundance of hundreds to thousands of analytes in thin tissue sections without requiring chemical labeling or antibodies (Jiang et al., 2023b). This capability allows for the precise mapping of small molecule spatial distributions and facilitates direct correlation with *in situ* pathological findings. Recent technological advancements have led to the development of key spatial metabolomics techniques, MALDI-MSI is a widely used technique for lipid detection, offering high sensitivity, rapid sample processing workflows, and excellent spatial resolution (Wan et al., 2025). Moreover, some studies have been carried out to explore atherosclerotic plaques from various aspects of atherosclerotic plaques using the TOF-SIMS(Lee et al., 2013; Lehti et al., 2015; Li et al., 2021), MALDI-MSI(Zaima et al., 2011; Martin-Lorenzo et al., 2015; Patterson et al., 2016; Visscher et al., 2019; Moerman et al., 2021), and DESI-MSI(Manicke et al., 2009; Li et al., 2023; Slijkhuis et al., 2023) techniques to explore atherosclerotic plaques from various aspects. With the development of MSI technology, which can target lesions more precisely for future studies, the rise of this technology has also successfully contributed to a new trend in the field of atherosclerosis research.

In this study, we used MALDI-MSI to investigate the spatial distribution of lipid metabolites within human carotid atherosclerotic plaques. By integrating multi-omics data, our experiments revealed that key genes involved in the production of AA-derived metabolites correlate with various inflammatory cells.

## 2 Materials and methods

### 2.1 Human tissue material and study design

The study protocol was approved by the Ethics Committee of the First Affiliated Hospital of Zhengzhou University (2024KY0120) and conducted in accordance with the Declaration of the Helsinki Ethical Principles. All participants were fully informed about the study, and following the thorough explanation of the study objectives and potential risks, all participants provided written informed consent. In the period spanning from October 2023 to March 2025, patient samples were collected from the Department of Endovascular Surgery at the First Affiliated Hospital of Zhengzhou University. The samples were obtained from patients who underwent carotid endarterectomy procedures. The diagnosis of patients with carotid atherosclerotic stenosis was made on the basis of preoperative imaging examinations (including ultrasound, CTA, and MRI) and postoperative pathological testing. Collection of plaque biological specimens was also undertaken. A total of 20 plaque samples were collected from 19 patients, based on the results of imaging and pathological analysis, and subsequently classified according to the American Heart Association’s classification of atherosclerosis (Stary et al., 1994). The study cohort was designed based on sample classification, dividing the samples into a stable plaque group (n = 10) and an unstable plaque group (n = 10). One representative plaque was selected from each of the stable and unstable groups for spatial imaging, these two sections were subjected to staining and immunohistochemical analysis alongside six newly acquired plaque sections (stable n = 3, unstable n = 3). Subsequently, fresh plaques were subsequently collected from six new patients (stable n = 1, unstable n = 5), with samples immediately subjected to single-cell sequencing analysis. In order to achieve this objective, metabolomics, spatial metabolomics, and single-cell transcriptomics data were integrated for analysis.

### 2.2 Sample preparation

After obtaining the specimen, we immediately rinse the surface impurities and blood with ice-cold physiological saline. Subsequently, the sample is rapidly frozen in liquid nitrogen for 5 min to fix its shape, and then transferred to a −80 °C freezer for subsequent use. Frozen tissue samples were fixed in three drops of distilled water during the cutting stage. The tissues were sectioned at 10 μm thickness using a Leica CM1950 cryostat (Leica Microsystems GmbH, Wetzlar, Germany) at −20 °C. Afterwards, the tissue se ctions were placed in groups on electrically conductive slides coated with indium tin oxide (ITO), and the slides with tissue sections were dried in a vacuum desiccator for 30 min. Consecutive sections were prepared from stable plaque sample and unstable plaque sample. A single section from each underwent MALDI-MSI acquisition, while adjacent sections were histologically stained with Oil Red O, Masson’s trichrome, Alizarin Red and H&E.

### 2.3 Immunostaining

Oil Red O, Masson’s trichrome, Alizarin Red S, H&E and other staining kits were purchased from Servicebio (Wuhan, China). The staining was performed according to the manufacturer’s protocol. Immunohistochemistry was conducted using CD68 (1:500) to visualize macrophages, α-smooth muscle actin (α-SMA) (1:1000) to visualize vascular Smooth Muscle Cells (SMCs). Immunofluorescence used ELOVL5 (1:500), ALOX5 (1:5000), CD86 (1:3000), CD163 (1:3000). Staining was performed on four samples per plaque type. The experimental methods are detailed in the Supplementary Material. Image analysis was performed using ImageJ software.

### 2.4 Metabolite extraction

The collected samples were thawed on ice, and metabolite were extracted with 80% methanol Buffer. Briefly, 50 mg of sample was extracted with 0.5 mL of precooled 80% methanol. The extraction mixture was then stored in 30 min at −20 °C. After centrifugation at 20,000 g for 15 min, the supernatants were transferred into new tube to and vacuum dried. The samples were redissolved with 100 μL 80% methanol and stored at −80 °C prior to the LC-MS analysis. In addition, pooled QC samples were also prepared by combining 10 μ L of each extraction mixture.

### 2.5 Parameter description

All samples were acquired by the LC-MS system followed machine orders. Firstly, all chromatographic separations were performed using an UltiMate 3000 UPLC System (Thermo Fisher Scientific, Bremen, Germany). An ACQUITY UPLC HSS T3 column (100 mm × 2.1 mm, 1.8 µm, Waters) was used for separation. The mobile phase consists of phase A (5 mmol/L ammonium acetate +5 mmol/L acetic acid + water) and phase B (acetonitrile). Gradient elution conditions were set as follows: 0–0.8 min, 2%–70%B; 0.8–2.8 min, 70%–90% B; 2.8–5.3 min, 90%–99% B; 5.3–5.9 min, 99% B; 5.9–7.5 min, 99%–2% B; 7.5–7.6 min, 2% B; 7.6–10.0 min, 2% B; The flow rate is 0.3 mL/min. The injection volume for each sample was 4 µL. The column oven was maintained at 40 °C.

A high-resolution tandem mass spectrometer Q-Exactive (Thermo Scientific) was used to detect metabolites eluted form the column. The Q-Exactive was operated in both positive and negative ion modes. Precursor spectra (70–1050 m/z) were collected at 70,000 resolution to hit an AGC target of 3e6. The maximum inject time was set to 100 ms. A top 3 configuration to acquire data was set in DDA mode. Fragment spectra were collected at 17,500 resolution to hit an AGC target of 1e5 with a maximum inject time of 80 ms. In order to evaluate the stability of the LC-MS during the whole acquisition, a quality control sample (Pool of all samples) was acquired after every 10 samples.

### 2.6 MALDI-MSI

Desiccated tissue sections mounted on ITO glass slides were sprayed using a matrix sprayer with 15 mg/mL DHB (2,5-dihydroxybenzoic acid), dissolved in 90%:10% (Acetonitrile: water). The sprayer (TM-Sprayer, HTX Technologies) temperature was set to 60 °C, with a flow rate of 0.12 mL/min, pressure of 8 psi. 25 passes of the matrix were applied to slides with 5 s of drying time between each pass. Matrix spraying instrument, moving speed: 1200 mm/min, track spacing: 3 mm.

MALDI timsTOF MSI experiments were performed on a prototype Bruker timsTOF flex MS system (Bruker Daltonics, Bremen, Germany) equipped with a 10 kHz smartbeam 3D laser. Laser power was set to 80% and then fixed throughout the whole experiment. The mass spectra were acquired in positive mode. The mass spectra data were acquired over a mass range from m/z 50–1300 Da. The imaging spatial resolution was set to 20 μm for the tissue, and each spectrum consisted of 400 laser shots. MALDI mass spectra were normalized with the Root Mean Square, and the signal intensity in each image was shown as the normalized intensity. MS/MS fragmentations performed on the timsTOF flex MS system in the MS/MS mode were used for further detailed structural confirmation of the identified metabolites. Mass spectrometry imaging data preprocessing was performed using SCiLS Lab MVS, 2025a Pro for RMS (root mean square) normalization. Metabolite spatial distribution maps were generated using R for min-max normalization and contrast enhancement (hotspot).

### 2.7 Single-cell sequencing analysis

Single-cell suspension was added to the 10x Chromium chip according to the instructions for the 10X Genomics Chromium Single-Cell 3'kit (V3), with the construction were performed according to standard protocols. Libraries were sequenced by LC-Bio Technology (Hangzhou, China) on an Illumina NovaSeq 6000 sequencing system (double-end sequencing, 150 bp) at a minimum depth of 20,000 reads per cell expectation of capturing 8,000 cells. cDNA amplification and library. Results from Illumina sequencing offline were converted to FASTQ format using bcl2fastq software (version 5.0.1). The scRNA-seq sequencing data were compared to reference genome using CellRanger software, and cellular and individual cellular 3'end transcripts were identified and counted in the sequenced samples. The output CellRanger expression profile matrix was loaded into Seurat (version 4.1.0) for filtering of low-quality cells from scRNA-seq data, and the filtered data was downscaled and clustered. Filtering low cell quality thresholds: number of genes expressed per cell >500, mitochondrial genes expressed in <25% of cells. Cells were projected into 2D space using t-SNE or UMAP These steps include: 1. calculating gene expression values using the LogNormalize method of Seurat’s “NormalizeData” function; 2. performing principal component analysis (PCA) using the normalized expression values, using the top 20 PCs for clustering, and Findcluster analysis; 3. analyze the marker genes of each cluster based on Findallmarker, and the marker genes were selected based on the following criteria: expressed in more than 10% of cells in each cluster, *p* value ≤0.01, gene expression ploidy logFC ≥0.26.

### 2.8 Statistical analysis

The acquired MS data pretreatments including peak picking, peak grouping, retention time correction, second peak grouping, and annotation of isotopes and adducts was performed using XCMS software. LC−MS raw data files were converted into mzXML format and then processed by the XCMS, CAMERA and metaX toolbox implemented with the R software. Each ion was identified by combining retention time (RT) and m/z data. Intensities of each peak were recorded and a three-dimensional matrix containing arbitrarily assigned peak indices (retention time-m/z pairs), sample names (observations) and ion intensity information (variables) was generated. The online KEGG, HMDB database was used to annotate the metabolites by matching the exact molecular mass data (m/z) of samples with those from database. If a mass difference between observed and the database value was less than 10 ppm, the metabolite would be annotated and the molecular formula of metabolites would further be identified and validated by the isotopic distribution measurements. We also used an in-house fragmentation spectrum library of metabolites to validate the metabolite identification. The PLS-DA analysis is performed by the R package ropls and the VIP values of each variable are calculated, perform 200 folds cross validation tests on the results to determine if the model is overfitting. Correlation analysis was performed by Pearson correlation coefficient of cor package. The three conditions of *p* Value <0.05, difference multiple >1.2 obtained by T-test and VIP calculated by PLS-DA analysis simultaneously met the screening of the final metabolites with significant differences. Hypergeometric-based enrichment analysis with KEGG Pathway was performed to annotate metabolite sequences, *p* Value <0.05. We isolated M1 and M2 macrophage subpopulations from single-cell transcriptomic data. Within each subpopulation, we calculated Spearman correlation coefficients between *ALOX5* and a set of classical proinflammatory genes (IL6, TNF, IL1B, etc.), followed by correlation heatmap and network analysis.

## 3 Results

### 3.1 Metabolomics exploration of metabolite heterogeneity in stable and unstable plaques

**FIGURE 1:**
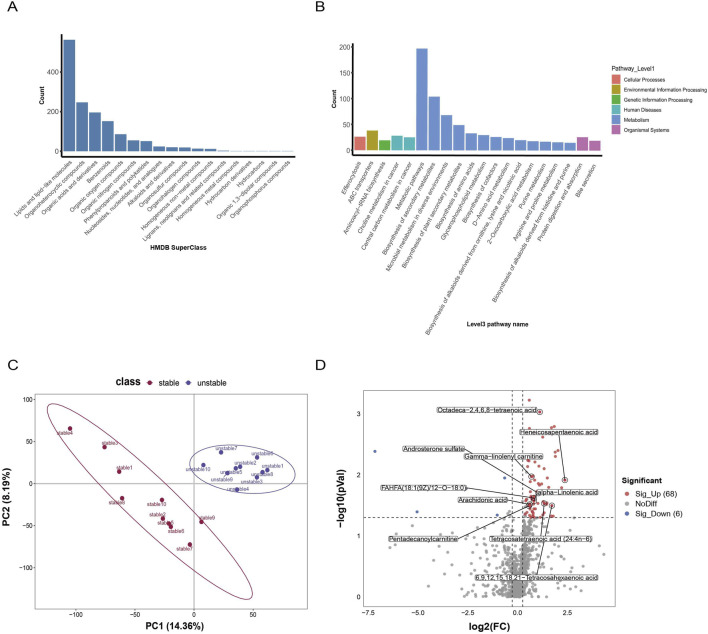
Metabolomic differences in human carotid artery atherosclerotic plaques. **(A)** HMDB annotation classification results for secondary metabolites in human carotid artery atherosclerotic plaques. **(B)** KEGG pathway annotations based on identified metabolites, showing the top 20 level 3 pathways by metabolite count. **(C)** Partial least squares discriminant analysis (PLS-DA) is conducted, with different groups represented by different colors. The relative positions of each point indicate the degree of dispersion among samples, with closer relative distances indicating more similar expression patterns. **(D)** Volcano plot showing up-regulated and down-regulated metabolites in stable and unstable plaques.

To characterize compositional differences and identify potential biomarkers in atherosclerosis, 20 carotid atherosclerotic plaques were collected from 19 patients. These were divided into stable (n = 10) and unstable (n = 10) groups. Through non-targeted identification with high confidence, we ultimately identified a total of 1463 metabolite components. We enhanced the classification annotation information for the identified metabolites using the HMDB database (Figure 1A) and the KEGG database. Based on the KEGG pathway annotations of the identified metabolites, we extracted the top 20 enriched KEGG pathways (Figure 1B). This enrichment revealed a significant proportion of lipid-related substances and various metabolism-associated pathways within the plaques. Subsequently, after performing quality control (QC) and normalization on all collected metabolite data, we conducted Partial Least Squares Discriminant Analysis (PLS-DA) to validate the reliability of the QC-processed data. Permutation tests of the model indicated that the slope of R^2^ was consistently greater than zero, while the slope of Q^2^ was consistently less than zero (Supplementary Figure S1A–C). This confirms that the PLS-DA model was not overfitted and validates the reliability of our results.

Next, to effectively screen for significant metabolites, we used PLS-DA for inter-group differential analysis. PLS-DA maximizes inter-group differences, achieving superior separation, it proved more adept at classification and at identifying features that distinguish the groups, revealing significant metabolic differences between stable and unstable plaques (Figure 1C; Supplementary Figure S1D). Using the criteria of FC ≥ 1.2, *p* < 0.05, and VIP ≥1, we identified 74 differentially abundant metabolites. Among these, 68 metabolites were upregulated in unstable plaques (Figure 1D). Our results demonstrate a substantial upregulation of differential lipids in unstable plaques, which aligns with findings from numerous previous studies on plaque vulnerability (Tomas et al., 2018; Wang et al., 2022).

### 3.2 Differential metabolites predict atherosclerotic plaque stability

**FIGURE 2:**
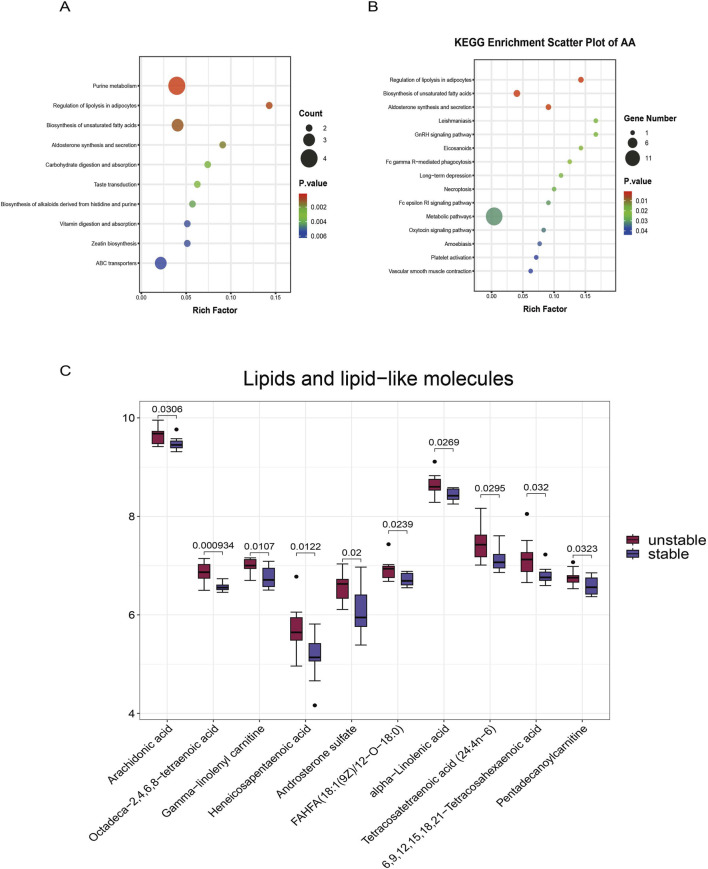
Metabolomics reveals the enrichment characteristics of arachidonic acid in plaques. **(A)** KEGG enrichment pathways of the top 10 differentially expressed metabolites. **(B)** KEGG functional enrichment analysis of AA. **(C)** Box plot quantification analysis of the top 10 upregulated metabolites in the ‘Lipids and lipid-like molecules’ category (*p* < 0.05).

We performed KEGG pathway enrichment analysis on the screened differential metabolites to predict the metabolic pathways involved in the atherosclerotic process based on the 74 identified metabolites. Among the 72 pathways detected, the top 10 is considered the most representative (Figure 2A). Interestingly, however, we found that AA was enriched in numerous pathways. AA-related pathways were both numerous and significant, being enriched in 22 out of the 72 pathways. The top 15 AA-enriched pathways were considered the most representative, particularly showing significant relevance in the Regulation of lipolysis in adipocytes, Biosynthesis of unsaturated fatty acids, Aldosterone synthesis and secretion, and Vascular smooth muscle contraction pathways (Figure 2B).

Subsequently, we classified the 68 significantly upregulated metabolites. Focusing specifically on the lipids and lipid-like molecules class (ranked within the top 10 classifications, *p* < 0.05; Supplementary Table S1) (Figure 2C). We further validated the predictive capability of the top 10 lipids for disease using ROC analysis (Supplementary Figure S2). We found that AA demonstrated specificity and sensitivity in plaques (AUC = 0.76, *p* = 0.04) (Supplementary Figure S2A), the ROC curve analysis further indicates that AA demonstrates good predictive ability for the disease. Furthermore, in the resulting differential volcano plot, AA was enriched among the significantly upregulated lipid metabolites (Figure 1D), indicating its correlation with plaque stability.

Collectively, our metabolomics data reveal that AA is involved in numerous key metabolic pathways relevant to atherosclerosis, exhibiting significant changes across different plaque types. This strongly suggests that AA plays a critical role in the pathogenesis and progression of the disease.

### 3.3 Spatial expression of AA in atherosclerotic plaque

Given the significant differential expression of AA between stable and unstable plaques, we employed MALDI-MSI for spatially resolved metabolomic analysis. The entire tissue section imaging area was selected to generate average mass spectra (Supplementary Figure S3A; Supplementary Figure S3B). Metabolite identification was achieved by matching acquired MS/MS spectra against integrated custom-built and public databases. In positive ion mode, we detected 1,546 metabolic features, with 516 metabolites confirmed through MS/MS fragmentation.

Within AA metabolism, its roles in immunomodulation, pro-inflammation, and inflammation resolution are highly significant (Tabas and Glass, 2013; Dennis and Norris, 2015). The initiation and resolution of inflammation are primarily controlled by local biological processes. Notably, substantial evidence links chronic inflammation directly to the emergence and progression of metabolic and cardiovascular diseases (Stables and Gilroy, 2011; Bennett and Gilroy, 2016; Merched et al., 2008). Various AA metabolites, particularly prostaglandins, leukotrienes (LTs) and thromboxanes, exhibit high bioactivity and contribute to the establishment of unresolved chronic inflammation surveillance. To summarize the above, we simultaneously conducted searches within our spatial omics data, and specifically selected Linoleic acid, AA, and Leukotriene D4 (LTD4) to characterize their spatial distributions within atherosclerotic plaques.

**FIGURE 3:**
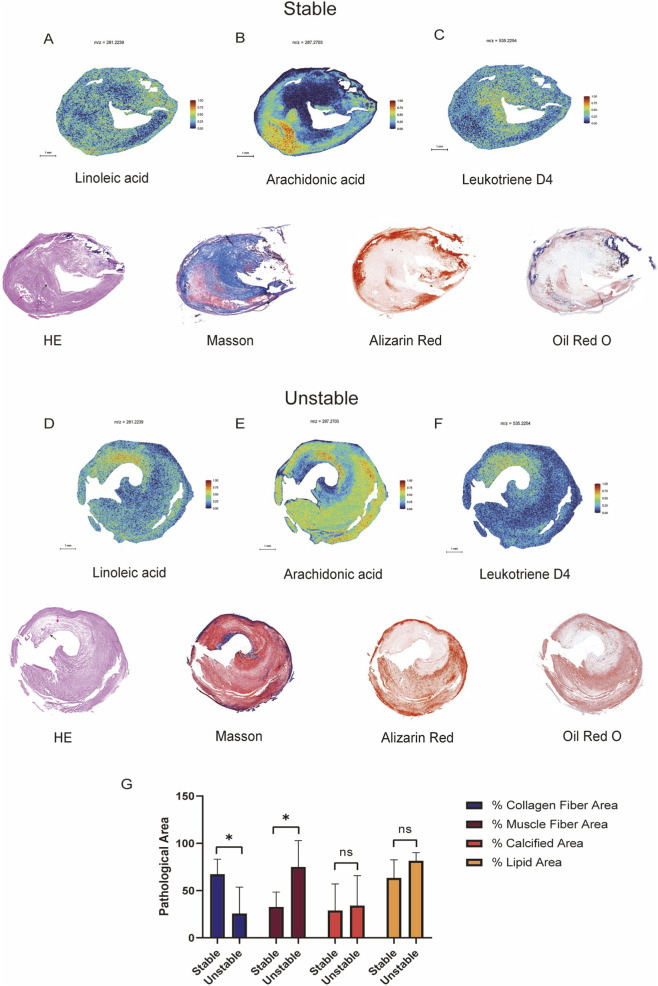
Localization and distribution of AA-related metabolites in spatial omics. Stable and unstable plaques were selected for imaging and then stained with Oil Red O, Masson's trichrome, Alizarin Red S, and H&E. Under MALDI(+)/DHB conditions, the several fatty acid-related ions, including linoleic acid signals ([M+H]^+^), arachidonic acid-related signals ([M+H-H_2_O]^+^), and leukotriene D4 signals ([M+K]^+^), were observed. Consecutive adjacent sections of the sample were stained and spatially imaged (MALDI-MSI): **(A,D)** Linoleic acid (m/z 281.2239), **(B,E)** AA (m/z 287.2703), **(C,F)** Leukotriene D4 (m/z 535.2254). **(G)** Pathological area of stable plaques (n = 4) and unstable plaques (n = 4), *: *p* < 0.05, ns: no significance. In H&E staining, the lipid core region (red arrow) and fibrous cap region (black arrow). scale bars:1 mm.

Under MALDI(+)/DHB conditions (Supplementary Figure S4; Supplementary Table S2), we observed several fatty acid-related ions (FA-related ions), including linoleic acid signals ([M + H]^+^), arachidonic acid-related signals ([M + H-H_2_O]^+^), and leukotriene D4 signals ([M + K]^+^). Spatial imaging revealed distinct distribution patterns of linoleic acid and arachidonic acid in the selected sample. In the MSI, linoleic acid was widespread in stable plaque (Figure 3A) but enriched in the lipid core of unstable plaque (Figure 3D). In contrast, AA exhibited a more surprising and localized distribution, primarily concentrating within the lipid core with diffusion into surrounding areas (Figure 3B,E). AA appeared concentrated within the plaque’s lipid core region, with diffusion into the surrounding areas. The degree of calcification within AA-enriched regions was markedly lower in the stable plaque compared to the unstable plaque. Furthermore, AA was found to be enriched within muscle fibers in both plaque types. LTD4 (Figure 3C,F) also showed differential distribution: in the plaque, it appeared enriched within the fibrous cap, and the calcification within its enrichment regions was not particularly evident.

Additionally, we quantified the areas corresponding to collagen fibers, muscle fibers, calcification, and lipids in two plaque types (each type n = 4), refining the compositional characterization of the plaques (Figure 3G). The differences were observed in the lipid core size, collagen fiber content, muscle fiber presence, and degree of calcification between stable and unstable plaques. Interestingly, our staining results confirmed that the fibrous cap in the stable plaque contained substantially more collagen compared to the unstable plaque. In the stable plaque, the lipid core was tightly encapsulated by fibrous tissue, making it less prone to rupture and contributing to enhanced structural integrity. Conversely, the large lipid pool in the unstable plaque elevates intramural stress, subjecting the thinner fibrous cap to greater mechanical strain and increasing its vulnerability to rupture (Zheng et al., 2005; Harman and Jørgensen, 2019; Babaniamansour et al., 2020).

### 3.4 The biological processes of AA are primarily mediated by macrophages

**FIGURE 4:**
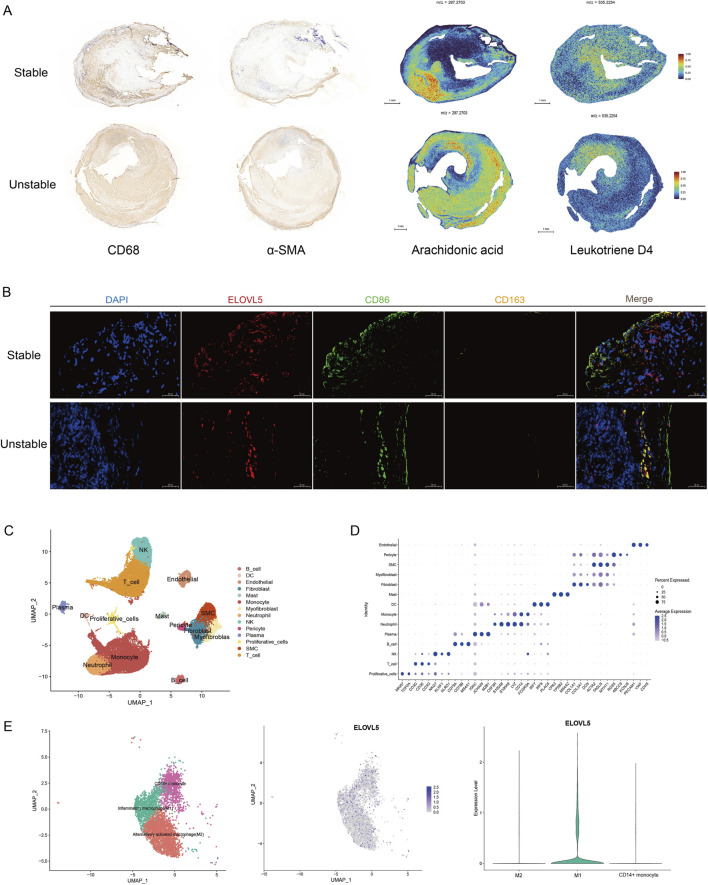
Spatial metabolomics and single-cell transcriptomics reveal a link between metabolite and macrophage functional states. **(A)** Spatial co-localization of CD68 macrophages, α-SMA smooth muscle cells with AA and Leukotriene D4. **(B)** Co-localization of *ELOVL5* with macrophages in human stable and unstable plaques by immunofluorescence. scale bars: 50 µm. UMAP plots **(C,D)** of single-cell sequencing data from patients with atherosclerosis. Re-clustering of cell clusters annotated as monocytes (4, 5, 8, 13, 20). Among them, monocytes are divided into three groups: Inflammatory macrophage (M1), Alternatively activated macrophage (M2), and CD14+ monocyte **(E)** Distribution of *ELOVL5* in key monocyte clusters.

Spatial metabolic imaging revealed a specific aggregation of AA across distinct plaque types. In light of AA’s pro-inflammatory properties and the substantial presence of inflammatory cells within plaques, we sought to determine if there was spatial co-localization. To explore this, we carried out immunohistochemical staining for CD68 and α-SMA on serial tissue sections. Comparative analysis of the sections revealed that AA exhibited strong co-localization with numerous macrophages, whereas its co-localization with SMCs was notably weaker (Figure 4A). Given the role of AA in inflammation and its specific association with macrophages, we next investigated whether these macrophages were polarized to a specific state. linoleic acid is converted into AA by *ELOVL5* (Hanna and Hafez, 2018). Upon cellular stimulation, such as during an inflammatory response, endogenous AA is released from membrane phospholipids (Dennis et al., 2011). For this purpose, we examined the relationship between *ELOVL5* expression (a key gene in AA synthesis) and macrophages. Interestingly, in our immunofluorescence co-staining, *ELOVL5* expression exhibited marked differences across macrophage polarization types. *ELOVL5* expression was significantly enriched in M1 macrophage regions, whereas enrichment in M2-dominant areas was minimal (Figure 4B). These findings appear to indicate that M1 macrophages promote *ELOVL5* to generate substantial amounts of AA, thereby driving inflammation.

To verify this view, we collected plaque samples from 6 patients for scRNA-seq. We identified a total of 22 cell clusters, which were classified and annotated into 14 distinct cell types, including B cells, Dendritic cells (DCs), Endothelial cells, Fibroblasts, Mast cells, Monocytes, Myofibroblasts, Neutrophils, Natural Killer cells (NK cells), Pericytes, Plasma cells, Proliferative cells, SMCs, and T cells (Figure 4C,D). We found that *ELOVL5* was enriched in a diverse array of cell clusters, including neutrophil cluster (3), monocyte clusters (5, 8, 13, 20), endothelial cluster (11), T cell clusters (12, 21), B cell cluster (14), proliferative cell cluster (16), and basal cell cluster (19) (Supplementary Figure S5A). Next, we subclustered the annotated monocyte cell clusters and annotated the resulting subpopulations based on gene signature scoring (Supplementary Figure S5B–D) (Supplementary Table S3), identifying three important monocyte subpopulations: Inflammatory macrophage (M1), Alternatively activated macrophage (M2), CD14^+^ monocyte (Figure 4E). Interestingly, *ELOVL5* is exclusively detected in M1 macrophages but is rarely enriched in M2 macrophages, which is consistent with our previous findings.

**FIGURE 5:**
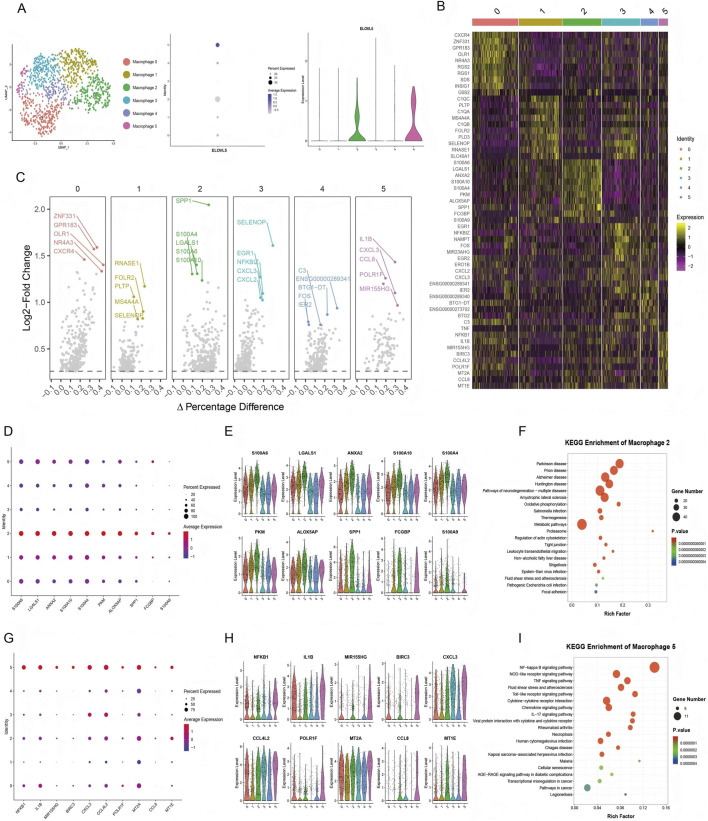
Association between M1 macrophage cluster and ELOVL5. **(A)** Re-clustering of M1 macrophages **(B)** top 10 feature genes with the highest upregulation factors in each M1 macrophage cluster. **(C)** Volcano plot of differential expression ratios for top 5 marker genes across cell populations. **(D,E)** Expression of the top 10 genes in Macrophage 2. **(F)** KEGG enrichment analysis of Macrophage 2 (top 20 pathways by *p*-value). **(G,H)** Expression of the top 10 genes in Macrophage 5. **(I)** KEGG enrichment analysis of Macrophage 5 (top 20 pathways by *p*-value).

To identify subclusters with the highest *ELOVL5* expression, we performed sub-clustering of M1 macrophages based on their proinflammatory profiles (Figure 5A–C). This analysis revealed six distinct macrophage subclusters, with *ELOVL5* most highly enriched in Macrophage 2 and Macrophage 5 (Figure 5A). Our findings indicate that *ELOVL5* serves as a key marker specific to these proinflammatory subpopulations, rather than being universally expressed across all M1 macrophages. In Macrophage 2, high *ELOVL5* expression correlated strongly with a pathogenic program driving tissue remodeling, as evidenced by co-expression of genes involved in inflammatory responses (*ANXA2*, *ALOX5AP*), cell migration/proliferation (*PKM*), and vascular remodeling (*LGALS1*, *S100A4*) (Figure 5D–F). In Macrophage 5, *ELOVL5* was associated with core inflammation signaling pathways, particularly the *NF-κB* pathway, which included upstream drivers (*NFKB1*, *IL1B*), effector cytokines/chemokines (*CXCL3*, *CCL4L2*, *CCL8*), and key regulators (*MIR155HG*) (Figure 5G–I). Collectively, these results suggest that *ELOVL5* may function as a critical hub connecting lipid metabolism with the dual pathogenic axes of inflammation and tissue remodeling, highlighting its potential value as a stage-specific therapeutic target.

### 3.5 Non-polarization-specific expression of ALOX5 in macrophages suggests a tuning role in inflammation

**FIGURE 6:**
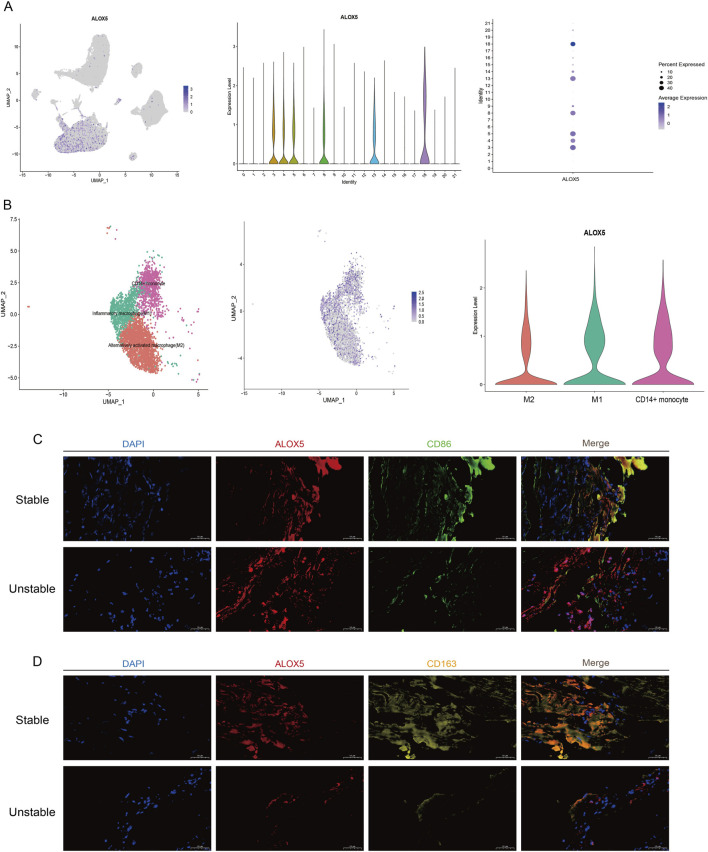
ALOX5 is enriched in multiple types of macrophages. **(A)** Cell clusters containing *ALOX5*. **(B)** Distribution of *ALOX5* in key monocyte clusters. **(C)** Co-localization of *ALOX5* with M1 macrophages in human stable and unstable plaques by immunofluorescence. scale bars: 50 µm. **(D)** Co-localization of *ALOX5* with M2 macrophages in human stable and unstable plaques by immunofluorescence. scale bars: 50 µm.

Our data revealed that LTs exhibit enrichment toward the fibrous cap region, particularly in unstable plaques, where their colocalization with CD68 was more evident (Figure 4A). Therefore, based on these findings, we investigated the macrophage polarization phenomenon by focusing on *ALOX5*, a key gene promoting AA-derived LTs production (Samuelsson, 1983). *ALOX5* is highly enriched in monocyte clusters (4, 5, 8, 13), as well as in neutrophil cluster (3) and mast cell cluster (18) in the sequencing data (Figure6A). In contrast to *ELOVL5*, *ALOX5* was expressed in both M1 and M2 macrophages (Figure 6B), indicating that its expression is not polarization-specific but rather ubiquitous across the macrophage lineage. To validate this, we performed immunofluorescence co-staining, which confirmed the view (Figure 6C,D).

**FIGURE 7:**
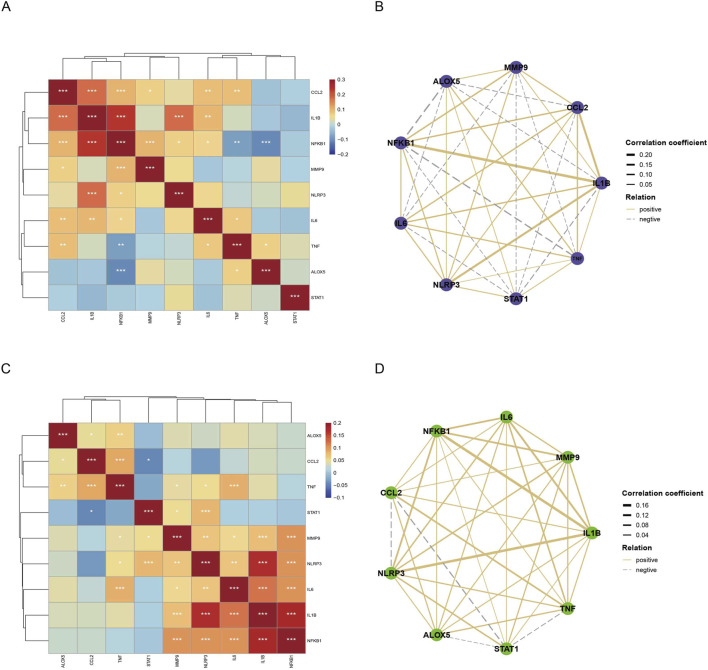
Correlation of ALOX5 Expression with Classic Inflammatory Genes. Correlation Heatmap **(A)** and Network Analysis **(B)** of *ALOX5* in M1 Macrophages. Correlation Heatmap **(C)** and Network Analysis **(D)** of *ALOX5* in M2 Macrophages. *: *p* < 0.05, **: *p* < 0.01, ***: *p* < 0.001.

To further define the role of *ALOX5* in macrophage polarization, we analyzed the co-expression of ALOX5 with classical pro-inflammatory markers in human plaques. In M1 macrophages, *ALOX5* expression showed the strongest positive correlation with *TNF* (*p* < 0.05) but, interestingly, a significant negative correlation with *NFKB1* (*p* < 0.001) (Figure 7A,B). This suggests that in M1 cells, the *ALOX5*-leukotriene pathway may not simply amplify inflammation but could fine-tune or periodically reinforce the intense inflammatory responses dominated by *NF-κB*. In contrast, pairwise correlation analysis in M2 macrophages revealed a general trend of *p*ositive correlations between *ALOX5* and most pro-inflammatory genes (Figure 7D). Among these, the correlations of *ALOX5* with *TNF* (*p* < 0.01) and *CCL2* (*p* < 0.05) were stronger than with other pro-inflammatory genes (Figure 7C). Our data indicate that even within the overall anti-inflammatory/reparative M2 context, specific inflammatory signals can drive *ALOX5* expression, enabling its participation in nuanced immune responses.

In summary, *ALOX5* serves as a pivotal node linking lipid metabolism and inflammatory responses, yet its ultimate contribution—whether pro-inflammatory or regulatory—likely depends on the cellular type and molecular environment in which it resides. This expression pattern of *ALOX5* highlights the functional heterogeneity and plasticity of macrophages within atherosclerotic plaques.

## 4 Discussion

Atherosclerosis is a chronic inflammatory disease characterized by prominent pathological features including endothelial dysfunction, inflammatory responses, and dysregulated lipid metabolism. However, the lesion tissues harbor abundant lipids of diverse composition and distinct functions. Whether the spatial distribution of these complex lipid components correlates with the structural stability of carotid atherosclerotic plaques, whether they exert critical pro--inflammatory or anti-inflammatory roles, or whether they significantly influence plaque remodeling and restructuring remains poorly understood and warrants comprehensive systematic investigation. This study integrates spatial metabolomics, untargeted metabolomics, and single-cell transcriptomics to delineate the spatial distribution and cellular drivers of key metabolites in human carotid atherosclerosis. Our findings reveal that AA and its derivatives are central players in plaque instability, with their spatial localization and cellular origins providing novel insights into disease pathogenesis and potential therapeutic targets.

Critically, we identified AA as significantly upregulated in unstable plaques compared to stable counterparts. This aligns with the established role of AA-derived eicosanoids (e.g., prostaglandins, LTs) as potent mediators of vascular inflammation and plaque progression (Dennis and Norris, 2015). Our spatial metabolomics data provide direct visual evidence that AA preferentially accumulates in lipid-rich core regions, zones classically associated with inflammatory activity and vulnerability. The colocalization of AA with macrophage-enriched areas suggests intimate crosstalk between lipid metabolism and innate inflammatory responses in driving plaque destabilization. Single-cell transcriptomics elucidated the cellular machinery governing AA metabolism. We found that key enzymes in AA biosynthesis and metabolism are predominantly expressed in macrophage subsets. Specifically, *ELOVL5* was enriched in inflammatory M1 macrophages, linking AA production to pro-inflammatory states. Conversely, *ALOX5* (critical for leukotriene synthesis) was expressed in both M1 and M2 macrophages, implicating it in both initiation and potential resolution of inflammation within plaques (Peng et al., 2025). AA pathways underscores macrophages as metabolic hubs coordinating lipid-mediated inflammation in atherosclerosis (Bennett and Gilroy, 2016). Furthermore, *ELOVL5* (which promotes AA synthesis) is associated with M1 macrophages. The *ALOX5* pathway within M1 cells is likely activated, converting AA into large quantities of LTs. This provides a highly specific and plausible molecular mechanism for how M1 macrophages drive inflammation. Moreover, our data reveal that lipid heterogeneity is not stochastic but organized within specific pathological niches (e.g., lipid core, fibrous cap, calcified regions), driven by specialized cell subsets. We found that the enzymatic machinery for AA metabolism is partitioned across functionally distinct macrophage subsets. The specific enrichment of *ELOVL5*—a key gene in AA synthesis—in pro-inflammatory M1 macrophages, particularly within pathogenic subclusters associated with tissue remodeling (Macrophage 2) and *NF-κB*-driven inflammation (Macrophage 5), reveals a direct genetic link. This suggests that M1 macrophages are not merely passive consumers of lipids but are metabolically programmed to fuel inflammation by autonomously amplifying the AA pool. In contrast, the expression pattern of *ALOX5*, the gateway enzyme for leukotriene production, presents a more complex picture. Its presence in both M1 and M2 macrophages indicates a broader, non-polarization-specific role. However, its context-dependent correlation patterns—negative with *NFKB1* in M1 yet positive with *TNF* and *CCL2* in M2—suggest that the functional output of *ALOX5* and its leukotriene products is not uniformly pro-inflammatory. Instead, it may fine-tune or modulate inflammatory responses in a manner dependent on the surrounding molecular milieu and cell state. This duality positions *ALOX5* as a potential regulatory node that could either exacerbate or temper inflammation.

## 5 Study strengths and limitations

To delineate AA-driven metabolic processes, we integrated bulk/spatial metabolomics with single-cell transcriptomics. Spatial metabolomics overcomes the limitations of low-dimensional studies by visually mapping lipid distributions (e.g., in necrotic cores, fibrous caps) for precise localization. Concurrently, single-cell analysis revealed the inflammatory cell-specific expression of key genes (*ELOVL5*, *ALOX5*). This confirms AA as the principal precursor for inflammatory mediators, which collectively govern the dynamics of inflammation.

The study elucidated the association between lipids and disease pathogenesis through omics approaches. Several limitations should be acknowledged. Our omics relatively small sample size and the inherent inter-individual heterogeneity of plaque specimens may introduce bias into the measurements, necessitating validation in larger cohorts. Furthermore, the spatial resolution of MALDI-MSI remains limited; however, ongoing technological advancements may bridge this gap in the future, potentially enabling metabolite localization at single-cell resolution, due to sample size limitations, the findings of this study should be regarded as preliminary and require validation through subsequent studies with larger sample sizes. While we proposed several plausible pathways and mechanisms, the specific signaling cascades and molecular targets require further elucidation. Future mechanistic studies are warranted to validate whether targeting AA metabolism can alter plaque stability *in vivo*.

Despite these limitations, this work provides a novel, integrated perspective on atherosclerotic plaque biology. We delineate how the spatial distribution of a key lipid (AA) is orchestrated by specific cell subsets with partitioned metabolic roles. This framework highlights spatially defined metabolic-inflammatory units within the plaque as critical determinants of disease progression.

## 6 Conclusion

In conclusion, by integrating spatial metabolomics with single-cell transcriptomics, this study provides a multidimensional atlas of lipid-driven inflammation in human carotid atherosclerosis. We demonstrate that AA is not only upregulated in unstable plaques but also spatially enriched within the macrophage-infiltrated lipid core, directly linking its distribution to plaque vulnerability. At the cellular level, we delineate a precise metabolic division of labor: the biosynthetic enzyme *ELOVL5* is specifically expressed in pro-inflammatory M1 macrophages, pinpointing this subset as a primary source of AA production. In contrast, the metabolizing enzyme *ALOX5* is ubiquitously expressed across macrophage lineages but exhibits context-dependent co-expression patterns—negatively correlated with *NF-κB* in M1 while positively correlated with key cytokines in M2 macrophages—suggesting a nuanced, fine-tuning role in inflammatory responses beyond simple pro-inflammatory signaling.

These findings transcend the limitations of single-omics approaches. We have successfully mapped specific lipid metabolites to their spatial pathological niches and deconvoluted the contributing cellular actors, thereby establishing a direct spatial-cellular-metabolic axis in plaque pathogenesis. This work not only deepens the mechanistic understanding of AA as a central node connecting lipid metabolism to inflammatory macrophage heterogeneity but also re-evaluate *ELOVL5* and *ALOX5* as potential stage-specific or cell-specific therapeutic targets for stabilizing vulnerable atherosclerotic plaques.

## Data Availability

The data presented in the study are deposited in the NCBI repository, accession number SAMN56357787, SAMN56357788, SAMN56357789, SAMN56357790, SAMN56357791, SAMN56357792.
