# Supplementary material for: Spatial localization of arachidonic acid in human carotid atherosclerotic plaques reveals a pro-inflammatory metabolic program in macrophages
Source: Front Mol Biosci. 2026 Mar 25;13:1786539. doi: 10.3389/fmolb.2026.1786539 (PMC13056667; doi:10.3389/fmolb.2026.1786539)
Supplement: Supplementary file 1 [file DataSheet2.pdf]

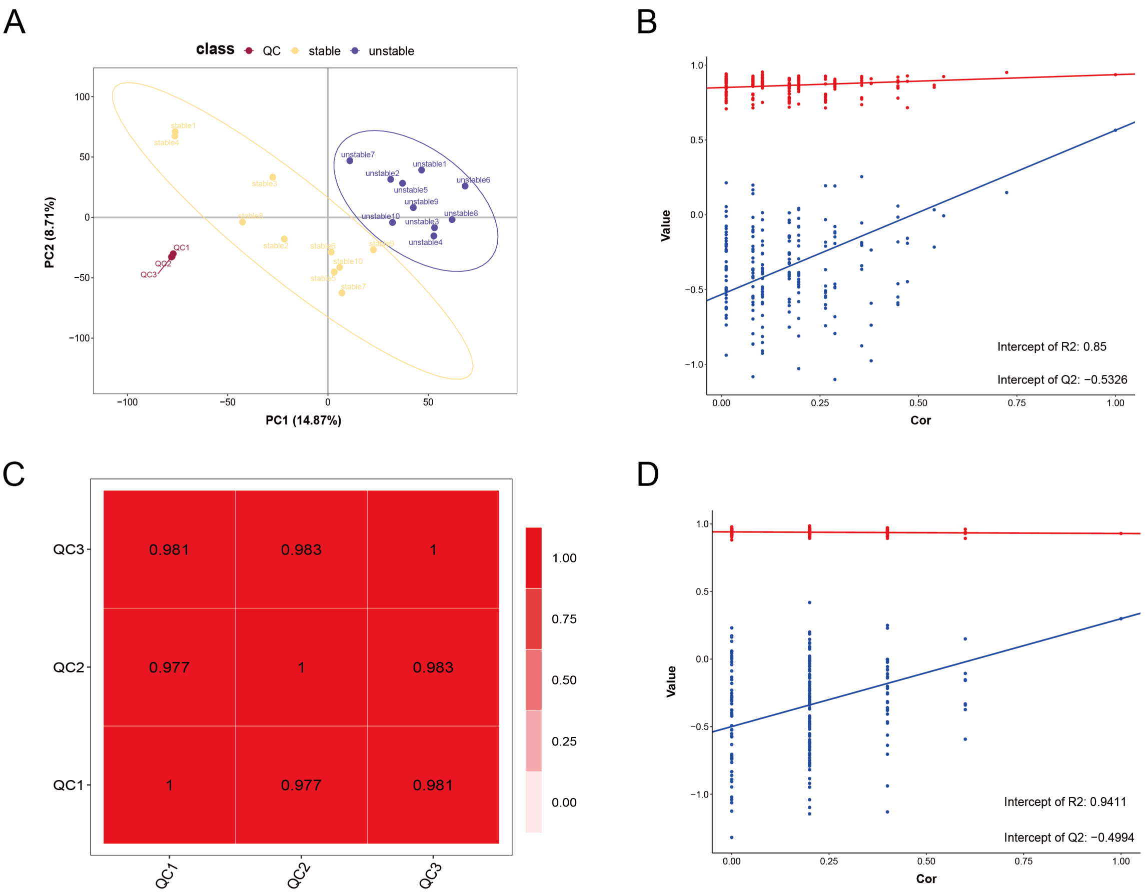
A
class QC stable unstable
PC1 (14.87%)
PC2 (8.71%)
stable1
stable4
stable3
stable2
stable7
stable10
unstable1
unstable2
unstable3
unstable4
unstable5
unstable6
unstable7
unstable8
unstable9
unstable10
QC1
QC2
QC3
B
Value
Cor
Intercept of R2: 0.85
Intercept of Q2: −0.5326
C
QC1
QC2
QC3
0.981 0.983 1
0.977 1 0.983
1 0.977 0.981
1.00
0.75
0.50
0.25
0.00
D
Value
Cor
Intercept of R2: 0.9411
Intercept of Q2: −0.4994

Figure S1. Partial Least Squares Discriminant Analysis (PLS-DA) and Permutation Test. (A) PLS-DA score plot: The horizontal axis represents the first principal component (PC1), and the vertical axis represents the second principal component (PC2). Each point in the plot represents a sample, with different groups shown in different colours. The relative positions of the points indicate the degree of dispersion among the samples; samples with closer relative distances indicate more similar expression patterns. Permutation test plots for the QC group **(B)** and non-QC group **(D)**: After randomly shuffling the group labels of each sample, modelling and prediction are performed. Each modelling corresponds to a set of R^2 and Q^2 values. Based on the Q^2 and R^2 values obtained after 200 shuffles and modelings, their regression lines can be obtained. In the figure, the R^2 regression line is red, and the Q^2 regression line is blue. When the x-coordinate is within [0,1], the R2 regression line is above the Q2 line, and the Q2 regression line intersects the y-axis at a point less than 0, this indicates that the model is not overfitted. **(C)** Pearson correlation coefficient analysis is performed on the abundance values of each QC sample after quality control, and the results are plotted. The higher the correlation between samples, the larger the value and the redder the colour. By observing the Pearson correlation analysis plots of each QC sample, we can assess the reproducibility of metabolite detection.

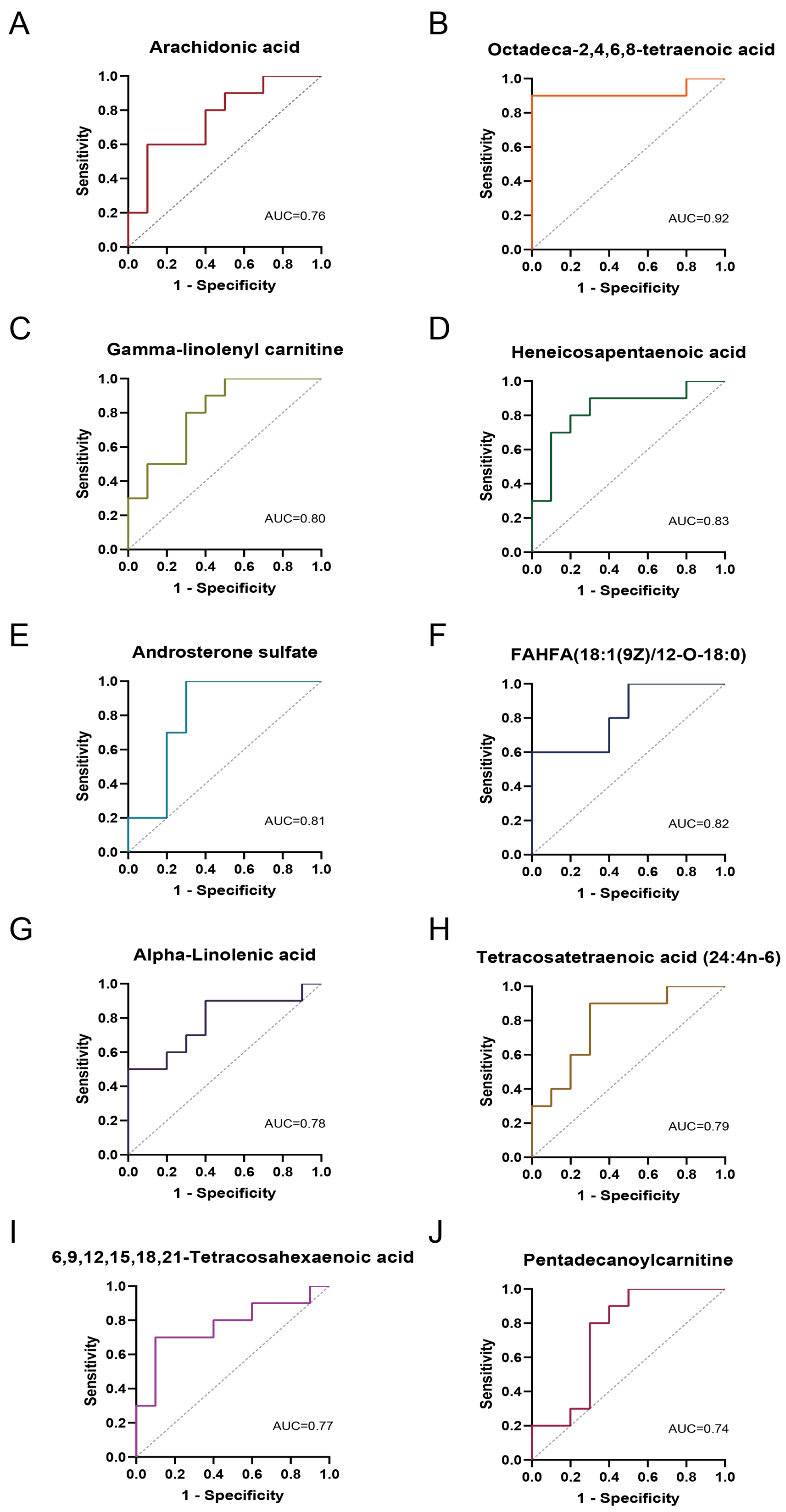
A
Arachidonic acid
AUC=0.76
B
Octadeca-2,4,6,8-tetraenoic acid
AUC=0.92
C
Gamma-linolenyl carnitine
AUC=0.80
D
Heneicosapentaenoic acid
AUC=0.83
E
Androsterone sulfate
AUC=0.81
F
FAHFA(18:1(9Z)/12-O-18:0)
AUC=0.82
G
Alpha-Linolenic acid
AUC=0.78
H
Tetracosatetraenoic acid (24:4n-6)
AUC=0.79
I
6,9,12,15,18,21-Tetracosahexaenoic acid
AUC=0.77
J
Pentadecanoylcarnitine
AUC=0.74
Sensitivity
1 - Specificity
0.0
0.2
0.4
0.6
0.8
1.0

Figure S2 The ROC of top10 lipids and lipid-like molecules.

(A) Arachidonic acid: AUC=0.76, 95%CI:0.54-0.97, *p*=0.04

(B) Octadeca-2,4,6,8-tetraenoic acid: AUC=0.92, 95%CI:0.76-1, *p*=0.0015

(C) Gamma-linolenyl carnitine: AUC=0.80, 95%CI:0.60-0.99, *p*=0.02

(D) Heneicosapentaenoic acid: AUC=0.83, 95%CI:0.63-1, *p*=0.01

(E) Androsterone sulfate: AUC=0.81, 95%CI:0.59-1, *p*=0.01

(F) FAHFA(18:1(9Z)/12-0-18:0): AUC=0.82, 95%CI:0.63-1, *p*=0.01

(G) Alpha-Linolenic acid: AUC=0.78, 95%CI:0.56-0.99, *p*=0.03

(H) Tetracosatetraenoic acid(24:4n-6): AUC=0.79, 95%CI:0.58-0.99, *p*=0.02

(I) 6,9,12,15,18,21-Tetracosahexaenoic acid: AUC=0.77, 95%CI:0.55-0.98, *p*=0.04

(J) Pentadecanoylcarnitine: AUC=0.74, 95%CI:0.50-0.97, *p*=0.06

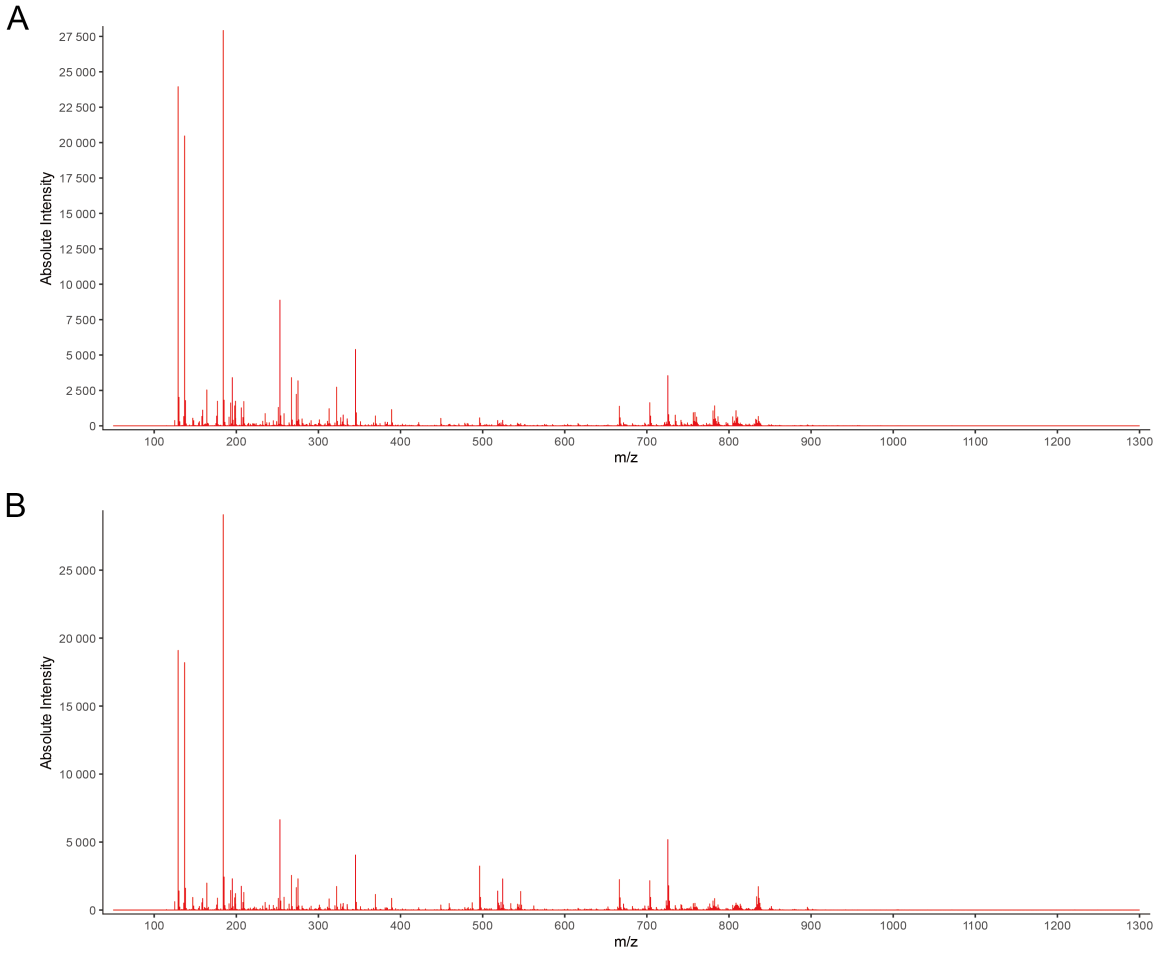
A
Absolute Intensity
m/z
B
Absolute Intensity
m/z

Figure S3. Average mass spectra of stable and unstable plaques. Import the raw imaging data into SCiLS Lab software for reading and perform root mean square standardisation. Select the entire imaging area of stable plaques **(A)** and unstable plaques **(B)** separately to obtain the average mass spectrum of each area, where the horizontal axis is the mass-to-charge ratio and the vertical axis is the average value of the peak intensity after root mean square standardisation in this area.

A

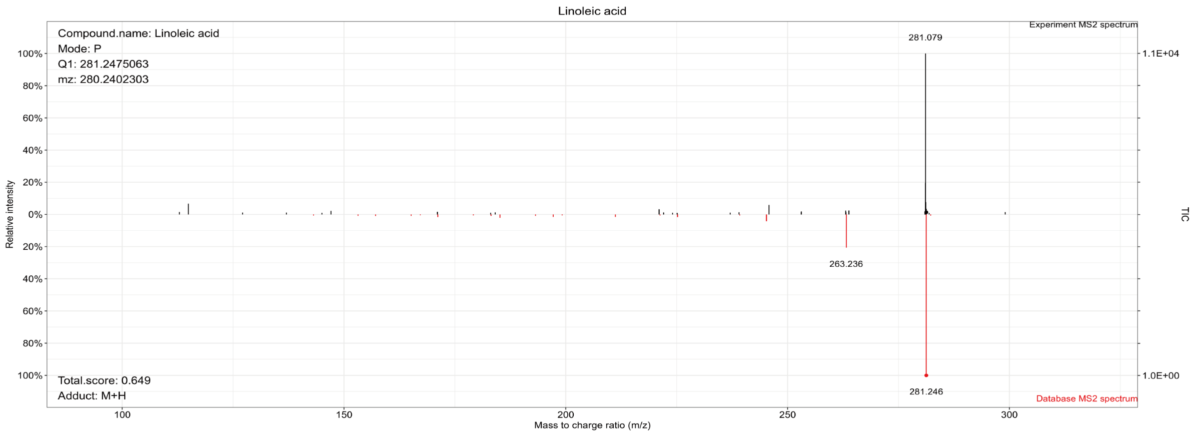
Linoleic acid
Compound.name: Linoleic acid
Mode: P
Q1: 281.2475063
mz: 280.2402303
Experiment MS2 spectrum
281.079
263.236
281.246
Total.score: 0.649
Adduct: M+H
Database MS2 spectrum
1.1E+04
1.0E+00
TIC
Relative intensity
Mass to charge ratio (m/z)

B

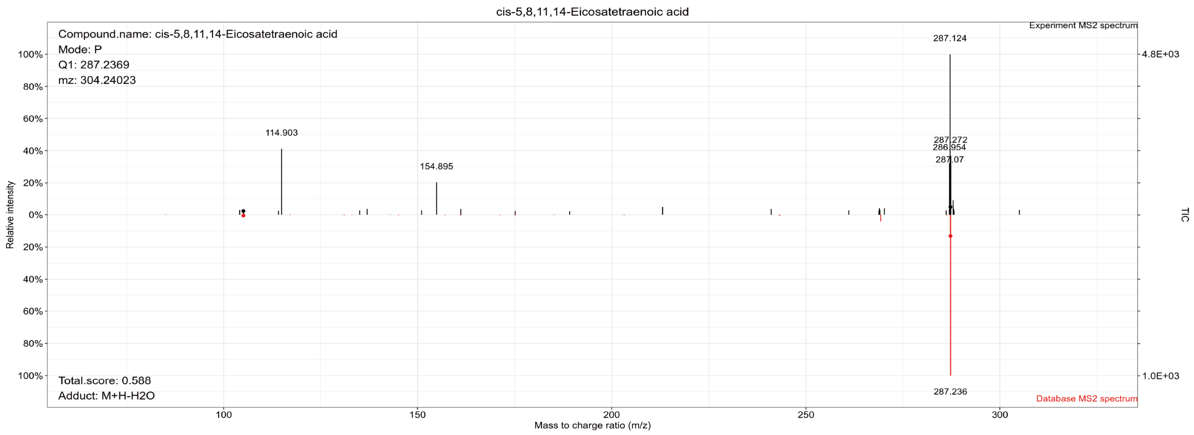
cis-5,8,11,14-Eicosatetraenoic acid
Compound.name: cis-5,8,11,14-Eicosatetraenoic acid
Mode: P
Q1: 287.2369
mz: 304.24023
Experiment MS2 spectrum
287.124
114.903
154.895
287.272
286.954
287.07
287.236
Total.score: 0.588
Adduct: M+H-H2O
Database MS2 spectrum
4.8E+03
1.0E+03
TIC
Relative intensity
Mass to charge ratio (m/z)

Figure S4. Secondary spectrum

(A) Secondary spectrum of linoleic acid.

(B) Secondary spectrum of arachidonic acid.

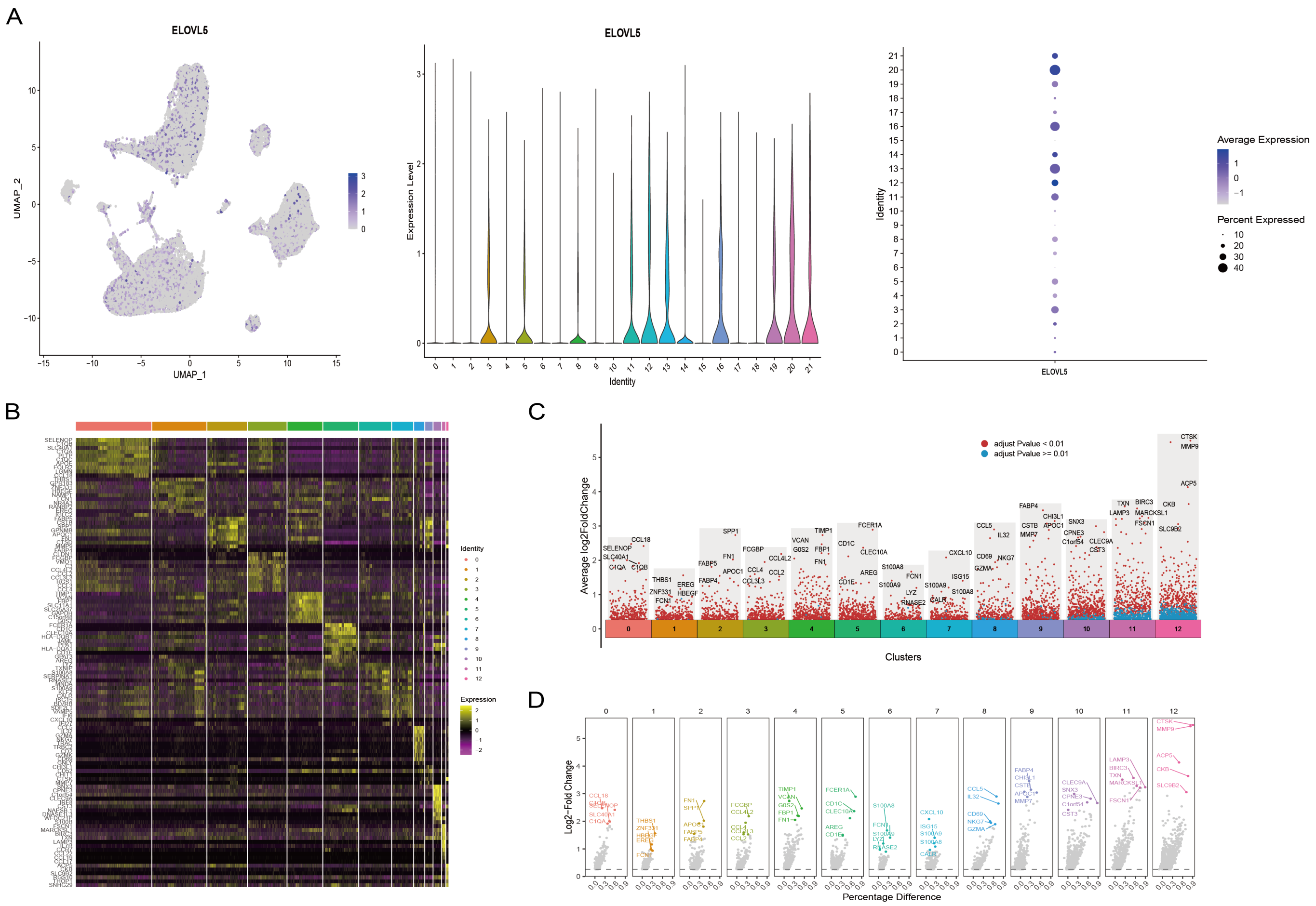
A
ELOVL5
UMAP_2
UMAP_1
ELOVL5
Expression Level
Identity
Identity
ELOVL5
Average Expression
Percent Expressed
B
Identity
Expression
C
adjust Pvalue < 0.01
adjust Pvalue >= 0.01
Average log2FoldChange
Clusters
D
Log2-Fold Change
Percentage Difference

Figure S5. Cell subpopulation analysis of macrophages. (A) Distribution of the *ELOVL5* in various cell clusters **(B)** Heatmap of gene expression in different cell populations, with the top 10 genes with the highest fold change in each cluster selected for heatmap visualization. **(C)** Volcano plot of the difference in fold change of characteristic genes among different cell populations, with the top 5 characteristic genes with the highest fold change selected for annotation. **(D)** Volcano plot of the difference in fold change and expression ratio of marker genes among different cell populations, with the top 5 characteristic genes with the highest fold change selected for annotation.
