# Supplementary material for: Spatial localization of arachidonic acid in human carotid atherosclerotic plaques reveals a pro-inflammatory metabolic program in macrophages
Source: Front Mol Biosci. 2026 Mar 25;13:1786539. doi: 10.3389/fmolb.2026.1786539 (PMC13056667; doi:10.3389/fmolb.2026.1786539)
Supplement: Supplementary file 2 [file DataSheet3.pdf]

Table S1. The top 10 of the lipids and lipid-like molecules

Name	Formula	HMDB ID	FC	p_value	q_value	VIP
Arachidonic acid	$C_{20}H_{32}O_2$	HMDB0001043	1.52	0.03	0.53	1.09
Octadeca-2,4,6,8-tetraenoic acid	$C_{18}H_{28}O_2$	HMDB0255898	2.16	0.001	0.20	1.71
Gamma-linolenyl carnitine	$C_{25}H_{43}NO_4$	HMDB0006318	1.62	0.01	0.40	1.31
Heneicosapentaenoic acid	$C_{21}H_{32}O_2$	HMDB0341326	5.19	0.01	0.42	2.17
Androsterone sulfate	$C_{19}H_{30}O_5S$	HMDB0002759	2.06	0.02	0.50	1.86
FAHFA(18:1(9Z)/12-O-18:0)	$C_{36}H_{68}O_4$	HMDB0112105	1.77	0.02	0.51	1.19
alpha-Linolenic acid	$C_{18}H_{30}O_2$	HMDB0001388	1.77	0.03	0.52	1.15
Tetracosatetraenoic acid (24:4n-6)	$C_{24}H_{40}O_2$	HMDB0006246	2.53	0.03	0.53	1.49
6,9,12,15,18,21-Tetracosahexaenoic acid	$C_{24}H_{36}O_2$	HMDB0013025	3.30	0.03	0.54	1.49
Pentadecanoylcarnitine	$C_{22}H_{43}NO_4$	HMDB0062517	1.49	0.03	0.54	1.06

Table S2. Fatty acid-related ions

Compounds	Formula	Precursor (Da)	Molecular weight (Da)	MS level	Adduct	CAS	HMDB	KEGG	Metlin	PubChem CID	Class I	Class II
Linoleic acid	C18H32O2	281.2239	280.2402304	2-Search-DB	M+H	60-33-3	HMDB0000673	C01595	191	5280450	FA	FFA
Arachidonic acid	C20H32O2	287.2703	304.2402304	2-Search-DB	M+H-H2O	506-32-1	HMDB0001043	C00219	193	444899	FA	FFA
Leukotriene D4	C25H40N2O6S	535.2254	496.2607079	1	M+K	-	-	-	96385	126456002	FA	Oxidized lipids

Table S3. Monocyte sub-clustering

cluster	Cell type	Cell Taxonomy ID	Matcged genes
0	Alternatively activated macrophage（M2）	CT:00000897	APOE, C1QA, C1QB, C1QC, CCL18, CD163, CD209, FOLR2, MERTK, MRC1, SELENOP, STAB1.
1	CD1C+ B dendritic cell	CT:00001463	AOAH, CSF3R, CSTA, EREG, F13A1, FCN1, FPR1, HBEGF, IL1B, IL1RN, NLRP3, VCAN.
2	Alveolar macrophage	CT:00000593	APOE, CXCL2, FABP4, FBP1, GPNMB, MARCO, MPP1, MSR1, SCD, TREM2.
3	Inflammatory macrophage（M1）	CT:00000870	CD74, CSF1R, DDX5, FCG3RA, FCGR1A, FCGR2A, HLA-DRA, IFNGR1, IL1B.
4	CD1C+ B dendritic cell	CT:00001463	ANXA1, CSF3R, CSTA, EREG, FCN1, FPR1, HBEGF, IL1B, IL1RN, VCAN.
5	Dendritic cell	CT:00000412	CD45.RA, CD86, CLEC10A, CST3, HLA-DPA1, HLA-DQA1, HLA-DRA, HLA-DRB1, ITGAX, LYZ2.
6	CD14-positive monocyte	CT:00001057	A100A9, CSF3R, FCN1, IER2, LGALS2, LYZ2, MNDA, S100A12, S100A8, VCAN.
7	Antigen presenting cell (RPS high)	CT:00000211	ARPC1B, ARPC2, ARPC3, ATP5MG, CFL1, FGL2, IFITM2, IFITM3, IGSF6, NPC2, SERF2.
8	T cell	CT:00000110	BCL11B, CCL5, CD2, CD3D, CD3E, CD3G, CD45.RA, CD6, CD69, CD7, CD8A, CD96, IL-7R, LCK, NKG7, SKAP1, SPOCK2, TRAC, TRAT1, TRBC2.
9	Adrenal gland inflammatory cell	CT:00002285	ATP5H, COX17, COX4I1, COX5B, COX6A1, COX6B1, MGLL, NDUFA11, SERF2, UQCR11.
10	CD141+ CLEC9a+ dendritic cell	CT:00001456	BTLA, CLNK, CSRP1, CYB5R3, DENND1B, DNASE1L3, DPP4, DUSP2, PRELID2, SLC24A4, VAV3, ZNF366.
11	Dendritic cell	CT:00000412	CD40, CD83, CD86, HLA-DPA1, HLA-DQA1, HLA-DRA, HLA-DRB1, IRF8.
12	Kidney intercalated cell	CT:00002179	ARL6IP5, ATP5H, ATP6AP2, C17ORF45, CKB, COL6A1, GLRX3, LAMTOR5, MTATP6P1, RPL35, UQCR11.
