# Supplementary material for: Spatial localization of arachidonic acid in human carotid atherosclerotic plaques reveals a pro-inflammatory metabolic program in macrophages
Source: Front Mol Biosci. 2026 Mar 25;13:1786539. doi: 10.3389/fmolb.2026.1786539 (PMC13056667; doi:10.3389/fmolb.2026.1786539)
Supplement: Supplementary file 3 [file DataSheet1.pdf]

SUPPLEMENTAL METHODS

Histological staining

H&E: The tissue sample is fixed, routinely embedded in paraffin, and sectioned. The sections are deparaffinized with xylene, hydrated through a graded ethanol series to water: xylene (I) for 5 min → xylene (II) for 5 min → 100% ethanol for 2 min → 95% ethanol for 1 min → 80% ethanol for 1 min → 75% ethanol for 1 min → rinsed with distilled water for 2 min. Staining with hematoxylin for 5-20 min (the duration can be adjusted based on staining results and requirements), followed by rinsing with tap water. Differentiation with differentiation solution for 30s. Rinsing with tap water for 15 min or warm water (approximately 50°C) for 5 min. Staining with eosin solution for 2 min. Rinsing with tap water. Routine dehydration, clearing, and mounting: 95% ethanol (I) for 1 min → 95% ethanol (II) for 1 min → 100% ethanol (I) for 1 min → 100% ethanol (II) for 1 min → xylene-phenol mixture (3:1) for 1 min → xylene (I) for 1 min → xylene (II) for 1 min → mounted with neutral resin.

Masson's trichrome: Dewaxing and hydration: The paraffin sections were immersed in sequence in Environmental Friendly Dewaxing Transparent Liquid I for 20min - Environmental Friendly Dewaxing Transparent Liquid II for 20min - Anhydrous ethanol I for 5min - Anhydrous ethanol II for 5min - 75% Ethyl alcohol for 5min, and then rinsed with tap water. Rewarming and fixing: The frozen sections were removed from the -20°C refrigerator and restored to room temperature, fixed with tissue fixating solution for 15min, and then rinsed with running water. The slices were soaked in Masson A overnight, rinse with tap water. Masson B and Masson C were prepared into Masson solution according to the ratio of 1:1. Then stain with Masson solution for 1 min, rinse with tap water. Differentiate with 1% hydrochloric acid alcohol for several seconds, rinse with tap water. Masson B and Masson C were prepared into Masson solution according to the ratio of 1:1. Then stain with Masson solution for 1 min, rinse with tap water. Differentiate with 1% hydrochloric acid alcohol for several seconds, rinse with tap water. Soak the slices in Masson D for 6 min, rinse with tap water; Masson E for 1 min; Do not wash the slides; slightly drain directly into Masson F for 2-30s. Rinse the slices with 1% glacial acetic acid and then dehydration with two cup of anhydrous
ethanol. Clearing and sealing: slides were soaked in 100% ethanol for 5 min; Xylene for 5 min; finally sealed with neutral gum.

Alizarin Red S: Place the paraffin sections in sequence in Xylene I for 20min - Xylene II for 20min - Anhydrous ethanol I for 5min - Anhydrous ethanol II for 5min - 75% Ethyl alcohol for 5min, washing with tap water. The slides were stained in Alizarin Red solution for 5 min, washed with tap water, and moved in the oven to dry. Put the sections into clean xylene transparent for 5min, sealing with neutral gum.

Oil Red O: Remove the frozen section from the -20°C refrigerator and restore it to room temperature, fix it with tissue fixing solution for 15min, wash with tap water, and dry. 6 parts of saturated oil red O dye solution and 4 parts of distilled water were fully mixed and homogenized, left to rest at 4°C overnight, filtered once with qualitative filter paper the next day, placed at 4°C for 24 hours and filtered again to

obtain oil red O working solution. Immerse the slices in the oil red dye solution for 8-10min (cover to avoid light). Take out the slices, stay for 3s, and then immerse in two cylinders of 60% isopropyl alcohol for differentiation, 3s and 5s respectively. The slides were
immersed in 2 tanks of pure water for 10s each. Take out the slides, stay for 3s, dip in hematoxylin for 3-5min and soak in 3 tanks of pure water for 5s, 10s and 30s respectively. The differentiation solution differentiated for 2-8s, 2 tanks of distilled water washed for 10s each, and the blue solution for 1s. The slides were gently immersed in 2 tanks of tap water for 5s and 10s each, and the staining
effect was checked by microscopy. Seal the slides with glycerin gelatin.

Immunohistochemistry
Air-dry frozen sections at room temperature, bake in a 37°C oven for 10-20 minutes, then fix in methanol for 20 minutes. Wash the slides in PBS (pH 7.4) on a decolorizing shaker 3 times, for 5 minutes each. Perform antigen retrieval as described in the table above. During this process, avoid excessive evaporation of the buffer; ensure the slides do not dry out. After natural cooling, wash the slides in PBS (pH 7.4) on a decolorizing shaker 3 times, for 5 minutes each. Incubate the sections in 3% hydrogen peroxide solution at room temperature, protected from light, for 25 minutes. Wash the slides in PBS (pH 7.4) on a decolorizing shaker 3 times, for 5 minutes each. Gently blot the slides dry after washing. Circle the tissue sections with a hydrophobic barrier pen. Apply 3% BSA or 10% normal rabbit serum within the circles to fully cover the tissue, and incubate at room temperature for 30 minutes for blocking. (Use 10% normal rabbit serum for blocking if the primary antibody is derived from goat; for primary antibodies from other species, use 3% BSA for blocking). Blot off the blocking solution. Apply the primary antibody diluted in PBS at an appropriate ratio onto the sections. Place the slides flat in a humidified chamber and incubate overnight at 4°C. Wash the slides in PBS (pH 7.4) on a decolorizing shaker 3 times, for 5 minutes each. Gently blot the slides dry. Apply the corresponding HRP-labeled secondary antibody (from the immunohistochemistry kit, matching the host species of the primary antibody) within the circles to cover the tissue. Incubate at room temperature for 50 minutes. Wash the slides in PBS (pH 7.4) on a decolorizing shaker 3 times, for 5 minutes each. Gently blot the slides dry. Apply freshly prepared DAB developing solution within the circles. Monitor the development time under a microscope; positive signal appears as a brown-yellow color. Rinse the slides with tap water to stop the development reaction. Counterstain with Hematoxylin for approximately 3 minutes, rinse with tap water, differentiate in hematoxylin differentiation solution for a few seconds, rinse with tap water, blue in hematoxylin bluing solution, and finally rinse under running tap water. Dehydrate and clear the sections by sequentially placing them in: 75% ethanol for 5 minutes, 85% ethanol for 5 minutes, 100% ethanol I for 5 minutes, 100% ethanol II for 5 minutes, n-butanol for 5 minutes, Xylene I for 5 minutes. Remove the slides from xylene and air-dry briefly. Mount with mounting medium.

Immunofluorescence

Fixation: Bake frozen sections at 37°C for 10-20 min. Fix in formaldehyde for 30 min. Wash in PBS (pH 7.4) on a shaker, 3 x 5 min. Antigen Retrieval: Perform antigen retrieval as required (prevent drying). Let cool naturally. Wash in PBS, 3 x 5 min. Peroxidase Blocking: Add 3% H_2O_2 and incubate in a light-proof humidified box for 25 min. Wash in PBS, 3 x 5 min. Serum Block: Incubate with a blocking serum for 30 min at room temperature in a humidified box. Use 10% rabbit serum if the secondary antibody is from goat. Use 3% BSA for secondary antibodies from other species. Primary Antibody: Remove block, add diluted primary antibody. Incubate at 4°C overnight in a humidified box. HRP Secondary Antibody: Wash in PBS, 3 x 5 min. Add the corresponding HRP-labeled secondary antibody. Incubate for 50 min at room temperature. TSA Detection: Wash in PBS, 3 x 5 min. Add the corresponding TSA reagent. Incubate for 10 min at room temperature in the dark. Wash in TBST, 3 x 5 min. Antibody Stripping: Add antibody stripping buffer to cover the tissue. Incubate at room temp for 5 min, then replace with fresh buffer and incubate at 37°C for 30 min. Wash in TBST, 3 x 5 min. DAPI Counterstain: Add DAPI staining solution. Incubate for 10 min at room temperature in the dark. Wash in PBS, 3 x 5 min. Autofluorescence Quenching: Add autofluorescence quenching agent for 5 min. Rinse with running water for 10 min. Mounting: Wash in PBS, 3 x 5 min. Let slides air-dry slightly, then mount with an anti-fade mounting medium. Image Acquisition: Image slides using the appropriate excitation/emission wavelengths for each fluorophore.
